# Fermi level tuning of Ag-doped Bi_2_Se_3_ topological insulator

**DOI:** 10.1038/s41598-019-41906-7

**Published:** 2019-03-29

**Authors:** Eri Uesugi, Takaki Uchiyama, Hidenori Goto, Hiromi Ota, Teppei Ueno, Hirokazu Fujiwara, Kensei Terashima, Takayoshi Yokoya, Fumihiko Matsui, Jun Akimitsu, Kaya Kobayashi, Yoshihiro Kubozono

**Affiliations:** 1 Research Institute for Interdisciplinary Science, Okayama University, Okayama, 700-8530 Japan; 2 Advanced Science Research Centre, Okayama University, Okayama, 700-8530 Japan; 3 Graduate School of Materials Science, Nara Institute of Science and Technology, Ikoma, 630-0192 Japan

## Abstract

The temperature dependence of the resistivity (*ρ*) of Ag-doped Bi_2_Se_3_ (Ag_x_Bi_2−x_Se_3_) shows insulating behavior above 35 K, but below 35 K, *ρ* suddenly decreases with decreasing temperature, in contrast to the metallic behavior for non-doped Bi_2_Se_3_ at 1.5–300 K. This significant change in transport properties from metallic behavior clearly shows that the Ag doping of Bi_2_Se_3_ can effectively tune the Fermi level downward. The Hall effect measurement shows that carrier is still electron in Ag_x_Bi_2−x_Se_3_ and the electron density changes with temperature to reasonably explain the transport properties. Furthermore, the positive gating of Ag_x_Bi_2−x_Se_3_ provides metallic behavior that is similar to that of non-doped Bi_2_Se_3_, indicating a successful upward tuning of the Fermi level.

## Introduction

Topological insulators are currently one of the most attractive research subjects in solid state physics because they are a new type of material characterized by gapless edge (surface) states and a finite energy gap^1–3^. These features are completely protected by time-reversal symmetry. The Bi compound Bi_1-x_Sb_x_ was the first to be confirmed as a ‘three-dimensional (3D) topological insulator’^4^, which had been theoretically predicted by Fu and Kane^5^. Since that discovery, many 3D topological insulators have been reported among Bi compounds^6–22^. Among these, Bi_2_Se_3_ has been extensively studied because its simple surface states consist of a single Dirac cone^6^. However, the electronic state of real Bi_2_Se_3_ single crystals as prepared is not so simple, since electrons are naturally accumulated due to the deficiency of Se^16–18^, *i.e*., the Fermi level reaches the conduction band of the bulk crystal, leading to difficulty in detecting surface states. Consequently, the Fermi level tuning is very important for topological insulator, Bi_2_Se_3_.

One way to solve this problem is the doping of Bi_2_Se_3_ (substituting another element for Bi). Ca doping of Bi_2_Se_3_ enabled the tuning of the Fermi level downward so that it approached the Dirac point^19,20^. Furthermore, Bi_2_Te_1.95_Se_1.05_ showed the insulating behavior characteristic of having the Fermi level lying on the Dirac point^9^. This ternary compound was followed by a quaternary compound, Bi_2−x_Sb_x_Te_3−y_Se_y_^21,22^. This compound’s Fermi level could be tuned by adjusting the value of x, which was determined by angle-resolved photoemission spectroscopy (ARPES)^22^; at x = 1.0 the Fermi level reached the Dirac point.

Thus, pursuing techniques for detecting surface states that are hidden by bulk states is important in the study of real single crystals of topological insulators. Furthermore, the Ca doping of Bi_2_Se_3_ described above leads not only to the tuning of the Fermi level but the scattering of carriers^20^. Therefore, various ways must be investigated to detect surface states without any additional damage. Thus, the successful discovery of new dopant for tuning the Fermi level is of significance, even if the Fermi level does not yet reach surface states. Furthermore, an upward tuning of the Fermi level for the electron-depleted (or hole-doped) Bi_2_Se_3_ is also important for the control of not only electronic structure but also electric transport. The Fermi level tuning by combination of metal doping and electrostatic carrier doping is an important way in materials design to induce novel physical properties.

In this study, we have doped Bi_2_Se_3_ with a small quantity of Ag atoms and characterized its transport properties in field-effect transistor (FET) structured devices. The transport properties were measured over a wide temperature range from 300 to 1.5 K, and the Hall effect was measured from 250 to 10 K to follow the transport properties.

## Results

### Characterization of Ag_x_Bi_2_Se_3_

**Figure 1:**
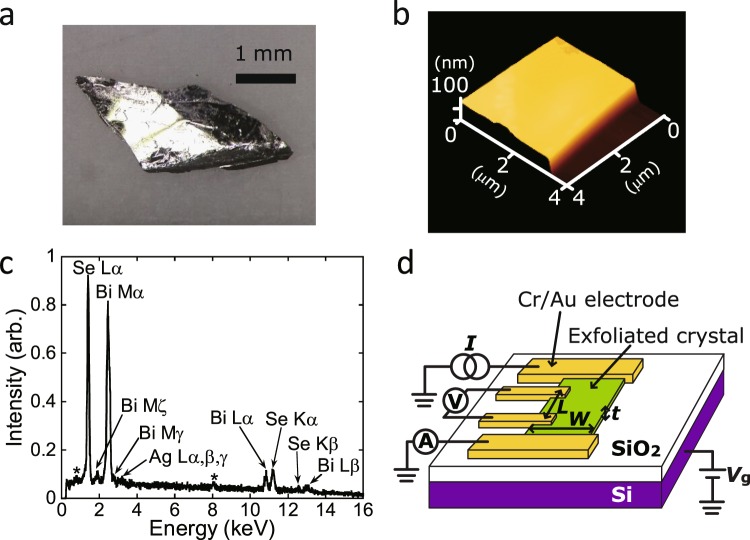
(**a**) Photograph of typical Ag_0.05_Bi_2_Se_3_ single crystal and (**b**) AFM image of exfoliated Ag_0.05_Bi_2_Se_3_ single crystal (135-nm thick). (**c**) EDX spectrum of Ag_0.05_Bi_2_Se_3_ single crystal. In (**c**), Cu Lα and Kα peaks with an asterisk originate from the Cu tape used for fixing the single crystal to the sample folder. (**d**) Schematic representations of Ag_0.05_Bi_1.95_Se_3_ single crystal FET with 300-nm thick SiO_2_ gate dielectric used for transport measurement.

Commercially available single crystals of Bi_2_Se_3_ were used for this study. The stoichiometry of one single crystal was checked by energy dispersive X-ray spectroscopy (EDX) (not shown), which provided the chemical composition of Bi_2_Se_2.55_. Also we evaluated the stoichiometry of other Bi_2_Se_3_ single crystals, showing that the chemical composition is expressed ‘Bi_2_Se_3.3(1)_’. Figure 1a and b show a typical optical image of Ag_0.05_Bi_2_Se_3_ single crystal, and an atomic force microscope (AFM) image of exfoliated Ag_0.05_Bi_2_Se_3_ single crystal (135-nm thick). The preparation of Ag_x_Bi_2_Se_3_ is described in Methods. The exfoliated single crystal was placed on a Si substrate with a 300-nm thick SiO_2_ gate dielectric. The EDX showed only peaks assignable to Ag, Bi, and Se atoms on the single crystal of Ag_0.05_Bi_2_Se_3_ (Fig. 1c). The averaged stoichiometry of the surface of five Ag_0.05_Bi_2_Se_3_ single crystals was determined to be Ag_0.11(1)_Bi_2_Se_3.2(6)_ in which the stoichiometry of Bi was fixed at 2, indicating doping with Ag atoms and an excess of Se. Here, it should be noticed that the scattering in stoichiometry of Se is too large, and the stoichiometry of Se in five single crystals is scattered from 2.1 to 3.8. The EDX of Ag_0.2_Bi_2_Se_3_ single crystals suggested that the chemical composition was expressed ‘Ag_0.2(1)_Bi_2_Se_3.3(3)_’ in which the stoichiometry of Bi was also fixed at 2, also demonstrating the Ag doping and an excess of Se atom, in the same manner as Ag_0.05_Bi_2_Se_3_. As described later, the electric transport for Bi_2_Se_3±δ_ single crystals with various Se amounts was similar to each other (metallic), while that was also same for Ag doped Bi_2_Se_3±δ_ single crystals (inverse V-shaped *R*–*T* plot).

Single crystal X-ray diffraction (XRD) also yielded information on the structure and chemical composition of Ag_0.05_Bi_2_Se_3_ crystal. Table S1 of Supplementary Information lists the crystallographic data of Ag_0.05_Bi_2_Se_3_. Furthermore, Bragg spots obtained from the XRD of Ag_0.05_Bi_2_Se_3_ single crystal are shown in Figure S1 of Supplementary Information, which exhibits clear Bragg spots without diffusing, indicating the high crystallinity of single crystal. The crystal structure was rhombohedral (space group: *R* $\bar{3}\,$m) which is consistent with that of Bi_2_Se_3_^23^. The lattice constants, *a* and *c*, were determined to be 4.146(6) and 28.66(4) Å, respectively, with being close to those of Bi_2_Se_3_ (4.18 and 28.7 Å)^23^. However, it is unclear whether Ag is not intercalated, because the lattice constant *c* does not so increase even in case of both the intercalation and the substitution for Bi^24,25^. Furthermore, the refinement of crystal structure also shows that no Ag atoms appear in any location other than the 6*c* and 3*a* sites which are occupied by Bi and Se, indicating that Ag may not be intercalated into the Bi_2_Se_3_ lattice. Namely, the difference Fourier map calculated from XRD for Ag doped Bi_2_Se_3_, where Ag, Bi and Se atoms are located at only the above sites, showed no electron density at other sites. Considering that Bi and Ag atoms become cations (Ag^+^ and Bi^3+^), Bi must be replaced by Ag, implying that hole is doped in Bi_2_Se_3_ by the replacement of Bi^3+^ with Ag^+^. Our recent experiment of X-ray fluorescence holography suggested that Ag was substituted for Bi, *i.e*., Ag_0.05_Bi_1.95_Se_3_^26^. Furthermore, the X-ray photoelectron holography provided the same conclusion^27^. As described later, hole accumulation due to Ag doping was further supported by the transport measurement. As a consequence, the chemical formula of Ag-doped Bi_2_Se_3_ can be expressed as ‘Ag_x_Bi_2−x_Se_3_’ where x is experimental nominal value, although the exact chemical formula is ‘Ag_x_Bi_2−x_Se_3±*δ*_’ owing to not only the partial replacement of Bi with Ag but also a deficiency or excess of Se. If considering the above EDX’s values (Ag_0.11(1)_Bi_2_Se_3.2(6)_ for Ag_0.05_Bi_2_Se_3_ and Ag_0.2(1)_Bi_2_Se_3.3(3)_ for Ag_0.2_Bi_2_Se_3_), the exact chemical formulae are re-expressed ‘Ag_0.10(1)_Bi_1.90_Se_3.0(6)_’ and ‘Ag_0.2(1)_Bi_1.8(1)_Se_3.0(3)_’, respectively, by considering a partial substitution of Bi with Ag, but we expressed as Ag_x_Bi_2−x_Se_3_ for nominal x through this paper. Exactly saying, it is unclear whether Se is deficient or excessive because of a large scattering of stoichiometry for Se, but the transport suggests deficiency as described later. Furthermore, a preliminary experiment of X-ray photoemission spectrum (XPS) of Ag_0.05_Bi_2_Se_3_ showed only a single peak ascribable to Ag^+^ (not shown)^27^, indicating that the valence of Ag is 1. To sum up, the information on the quality of Ag doped Bi_2_Se_3_ single crystals obtained from these studies guarantees that the used single crystal of Ag_x_Bi_2−x_Se_3_ is sufficiently available for the study on transport properties of hole doped (or electron-depleted) Bi_2_Se_3_.

### Transport properties of Ag_0.05_Bi_1.95_Se_3_

**Figure 2:**
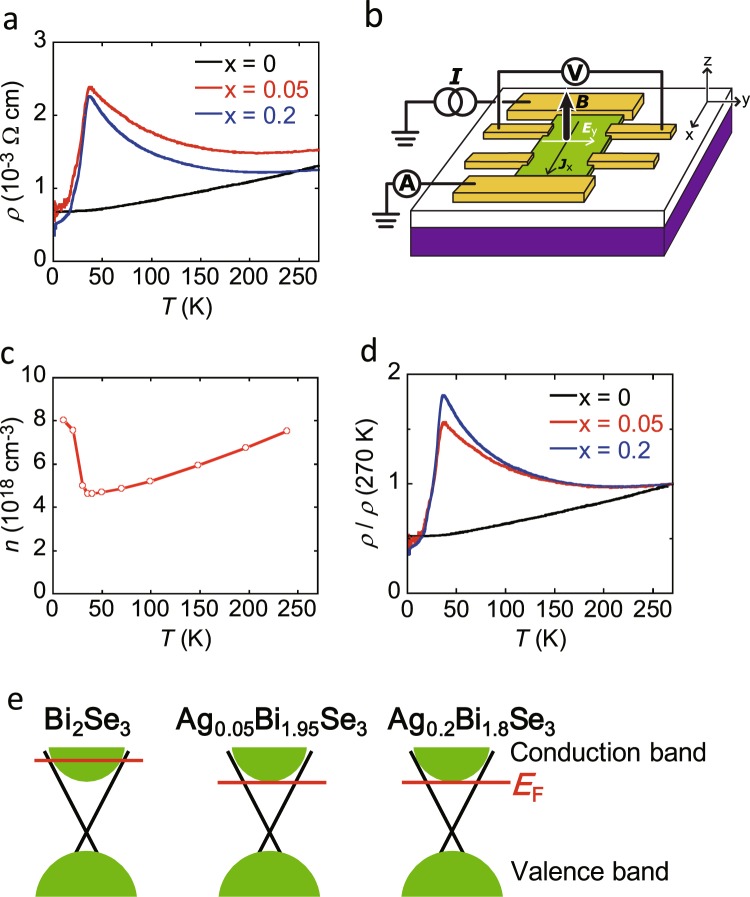
(**a**) *ρ* – *T* plots of single crystals of Bi_2_Se_3_ (60-nm thick), Ag_0.05_Bi_1.95_Se_3_ (105-nm thick) and Ag_0.2_Bi_1.8_Se_3_ (110-nm thick). (**b**) Device structure used for measuemnt of Hall effect. *J*_x_ refers to current density along x direction, and *E*_y_ refers to electric field along y direction. Here, *B* is applied along z direction. (**c**) *n*–*T* plot of Ag_0.05_Bi_1.95_Se_3_ (82-nm thick) determined from Hall effect measurement. (**d**) *ρ*/*ρ*(270 K) – *T* plots of single crystals of Bi_2_Se_3_ (60-nm thick), Ag_0.05_Bi_1.95_Se_3_ (105-nm thick) and Ag_0.2_Bi_1.8_Se_3_ (110-nm thick). (**e**) Schematic representations of electronic structures in Bi_2_Se_3_, Ag_0.05_Bi_1.95_Se_3_, and Ag_0.2_Bi_1.8_Se_3_. All transport properties were measured in four-terminal measurement mode.

Figure 1d shows the structure of an Ag_0.05_Bi_1.95_Se_3_ single-crystal FET with a 300-nm thick SiO_2_ gate dielectric. The transport properties were measured using this device structure. The values of resistivity (*ρ* (=*RWt*/*L*), sheet resistivity (*ρ*_s_ (=*RW*/*L*)) and sheet conductance (*σ*_s_ (=1/*ρ*_s_)) were evaluated from the slope (or *R*) derived by the linear fitting of the *V*–*I* plots in four-terminal or two-terminal measurement mode where *R*, *L*, *W*, and *t* are resistance, channel length, channel width, and thickness of the single crystal, respectively, shown in Fig. 1d. Details of measurement are described in Methods, and the measurement mode for each graph (transport property) is described in each Figure caption. The *ρ* (=*RWt*/*L*) of Ag_0.05_Bi_1.95_Se_3_ single crystal (105-nm thick) is plotted as a function of temperature (*T*), showing a clear transition at 35 K (Fig. 2a). The *ρ* of Ag_0.05_Bi_1.95_Se_3_ (x = 0.05, Fig. 2a) first increases with decreasing temperature, then rapidly decreases below 35 K, showing an inverse V-shaped *ρ*–*T* plot. This behavior is quite unlike the *ρ*–*T* plot of non-doped Bi_2_Se_3_ single crystal (60-nm thick, x = 0 in Fig. 2a), which exhibits simple metallic behavior. The observation of such metallic behavior implies that the transport properties reflect the conduction band because the Fermi level crosses the conduction band. As seen from Fig. 2a, the *ρ* of Ag_0.05_Bi_1.95_Se_3_ is higher than that of Bi_2_Se_3_. Thus, the transport properties of Ag_0.05_Bi_1.95_Se_3_ show that the Fermi level shifts downward in the conduction band.

As described in the section of characterization of this paper, the *ρ*–*T* plot was always the same (metallic) in the Bi_2_Se_3±δ_ single crystals with different Se amounts used in this study, and that of Ag doped Bi_2_Se_3±δ_ was also the same (inverse V-shaped *ρ–T* plot), guaranteeing that the reliable comparison in electric transport between Bi_2_Se_3_ and Ag doped Bi_2_Se_3_. From this study, the inhomogeneity of Se within δ seems not to affect the transport property. Namely, we can discuss only the effect of Ag doping on transport property in this study.

### Carrier density of Ag_0.05_Bi_1.95_Se_3_

To pursue this inverse V-shaped *ρ*–*T* plot more precisely, we measured Hall effect of 82 nm-thick Ag_0.05_Bi_1.95_Se_3_ single crystal; the device structure used for Hall effect measurement is shown in Fig. 2(b). Hall effect was measured by applying perpendicular magnetic field *B* to the substrate from −1 to 1T. Hall resistance, *R*_yx_, was a linear function of *B* in this range, and Hall coefficient *R*_H_ was obtained from *R*_H_ = *t dR*_yx_/*dB*. The *R*_H_ shows carrier type and carrier density because of *R*_H_ = -(*ne*)^−1^ for electron and *R*_H_ = (*ne*)^−1^ for hole, where *n* (>0) and *e* (>0) refer to carrier density and elementary charge, respectively; the *R*_H_ is given in SI unit. The observed *R*_H_ value was negative, showing that the carrier is still electron. Figure 2c shows *n*–*T* plot, which completely provides the V-shaped structure. This is consistent with the *ρ*–*T* plot. Here, we must stress that the inverse V-shaped *ρ*–*T* plot is completely reproduced in the sample (82-nm thick Ag_0.05_Bi_1.95_Se_3_ single crystal) used for the Hall effect measurement. As a consequence, the increase in *ρ* can be well explained by the decrease in *n*, and the *n* value exactly becomes minimum at the transition temperature, *T*_cr_, in *ρ*–*T* plot (*T*_cr_ = 35 K in Fig. 2a). This unambiguous consistence means that the *ρ*–*T* plot reflects the variation of electron density against temperature. Here, we must comment on the *n* value for Ag_0.05_Bi_1.95_Se_3_. The minimum *n* is 4.7 × 10^18^ cm^−3^ at 35 K, which corresponds to the carrier density per area, *n*_s_ (=*nt*), of 3.8 × 10^13^ cm^−2^. The value of the minimum *n*_s_ further exceeds ~1 × 10^13^ cm^−2^ which is the maximum density for pure surface conduction in Bi_2_Se_3_^12,28^. Therefore, the *n* meaningfully includes bulk carrier density in the conduction band at all temperatures. This suggests that the decrease in the resistivity below *T*_cr_ originates from the increase in bulk conductivity.

Recently, the variability of the transport properties of Sb-doped Bi_2_Se_3_ was investigated in FET structured devices^12^, showing metallic behavior in non-doped Bi_2_Se_3_ and non-metallic behavior in 6–7% Sb-doped Bi_2_Se_3_. The *ρ*–*T* plot of Ag_0.05_Bi_1.95_Se_3_ (Fig. 2a) is similar to the *R*_s_ - *T* of 6–7% Sb-doped Bi_2_Se_3_; *R*_s_ is sheet resistance (*R*_s_ = *RW*/*L* = *ρ*_s_). Moreover, the *R*_s_ of Sb-doped Bi_2_Se_3_ increased by an order of magnitude^12^. The behavior is similar to the case of Ag-doped Bi_2_Se_3_ (Fig. 2a), although the ratio of increase is less than twice. As suggested for Sb-doped Bi_2_Se_3_^12^, the Ag doping of Bi_2_Se_3_ must reduce the contribution from bulk conductivity. However, there are points of difference between Ag_0.05_Bi_1.95_Se_3_ and 6–7% Sb-doped Bi_2_Se_3_: (1) the *T*_cr_ is different (35 K for Ag_0.05_Bi_1.95_Se_3_ and 100 K for Sb-doped Bi_2_Se_3_), and (2) for Sb-doped Bi_2_Se_3_, saturation is observed below 50 K, rather than a rapid decrease in *R*_s_ below *T*_cr_. In addition, application of positive and negative *V*_g_ to Sb-doped Bi_2_Se_3_ produced a rapid decrease in *R*_s_ below *T*_cr_^12^. Therefore, it is suggested that the Fermi level in Ag_0.05_Bi_1.95_Se_3_ lies farther from the Dirac point than in 6–7% Sb-doped Bi_2_Se_3_.

**Figure 3:**
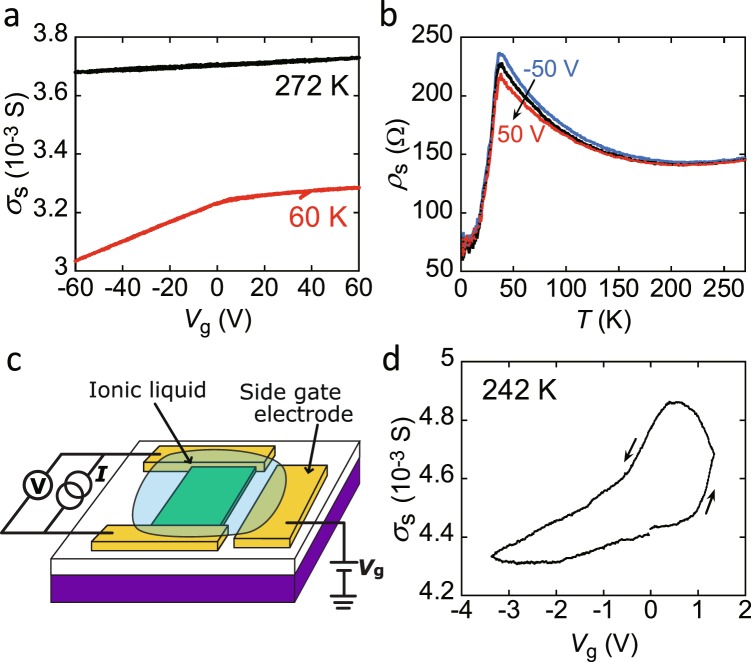
(**a**) *σ*_s_–*V*_g_ plots at 272 and 60 K and (**b**) *ρ*_s_–*T* plots of Ag_0.05_Bi_1.95_Se_3_ single-crystal FET with 300-nm thick SiO_2_ gate dielectric; thickness of single crystal is 105 nm. In (**b**), *V*_g_ values are fixed to −50, 0, and 50 V. In (**a**) and (**b**), transport properties are measured in four-terminal measurement mode. (**c**) Schematic representations of Ag_0.05_Bi_1.95_Se_3_ single crystal EDLT with bmim[PF_6_] ionic liquid used for transport measurement. (**d**) *σ*_s_–*V*_g_ plot of Ag_0.05_Bi_1.95_Se_3_ single crystal EDLT at 242 K; thickness of single crystal is 105 nm. In (**d**), transport properties are measured in two-terminal measurement mode.

Considering transport property of Sb-doped Bi_2_Se_3_^12^ and the *n* determined from Hall effect, we concluded that the Fermi level of Ag_0.05_Bi_1.95_Se_3_ probably lied on the bottom area of conduction band or the top of surface states. Admittedly, preliminary ARPES suggests the crossing of the Fermi level to the bottom of conduction band (not shown); the ARPES of this sample changed gradually under the irradiation of light during the measurement. Thus, the ARPES study must be more precisely examined because the ARPES easily varies under various conditions^29^. Moreover, the transfer plot at 60 K shown in Fig. 3(a) exhibited different slope above/below *V*_g_ = 0 V, indicating that the Fermi level is located near the bottom of conduction band at *V*_g_ = 0 V. This result significantly supports the Fermi level’s location near bottom of the conduction band.

To sum up, the location of the Fermi level determined from Hall effect and comparison between transport properties in Ag doped Bi_2_Se_3_ (our study) and Sb doped Bi_2_Se_3_ (ref.^12^) is consistent with that determined from the preliminary ARPES and the FET data (Fig. 3(a)). Therefore, the transport data (*R*–*T* plot and *n*–*T* plot) achieved in this study is complementary for the ARPES and FET data. In addition, the Hall effect measurements of Bi_2_Se_3_ and possible more hole-doped Bi_2_Se_3_ such as Ag_0.2_Bi_1.8_Se_3_ may be necessary for pursuing exact position of the Fermi level and for understanding the transport properties. These are future tasks.

### Transport properties of Ag_0.2_Bi_1.8_Se_3_

Figure 2a shows the temperature dependence of *ρ* in Ag_0.2_Bi_1.8_Se_3_ single crystal (110-nm thick, x = 0.2 in Ag_x_Bi_2−x_Se_3_). The *ρ*–*T* plot for Bi_2_Se_3_ (metallic behavior) is changed to the inverse V-shape by Ag doping. The ratio *ρ*/*ρ*-at-270-K (abbreviated as *ρ*(270 K)) shows almost the same behavior above 100 K between Ag_0.2_Bi_1.8_Se_3_ and Ag_0.05_Bi_1.95_Se_3_ (Fig. 2d), implying that the Fermi level is located at almost the same position. At 35–100 K, the *ρ*/*ρ*(270 K) was slightly higher in Ag_0.2_Bi_1.8_Se_3_ than that in Ag_0.05_Bi_1.95_Se_3_. The *T*_cr_ was also the same between x = 0.05 and 0.2. Furthermore, the *ρ* value in Ag_0.2_Bi_1.8_Se_3_ above *T*_cr_ is slightly smaller than that in Ag_0.05_Bi_1.95_Se_3_ (Fig. 2a). These results indicate that the larger amount of Ag doping than x = 0.05 in Ag_x_Bi_2−x_Se_3_ is not so effective to tune the Fermi level downward, indicating no hole doping at more than 0.1 (=0.05 × 2), *i.e*., no substitution of Ag for Bi. Recent photoelectron holography measurement suggests the occurrence of not only substitution but also intercalation for x = 0.2^27^, although only a substitution is suggested for x = 0.05^26,27^, indicating both electron and hole doping for Ag_0.2_Bi_1.8_Se_3_. If it is the case, the low *ρ* in Ag_0.2_Bi_1.8_Se_3_ may be reasonably explained, because electron accumulation cancels the effect of hole doping. Schematic representations of the suggested electronic states of Ag_x_Bi_2−x_Se_3_ (x = 0.05 and 0.2) and Bi_2_Se_3_ are also shown in Fig. 2e.

### Electrostatic carrier doping of Ag_0.05_Bi_1.95_Se_3_

Figure 3a shows *σ*_s_ as a function of *V*_g_ of −60 to 60 V recorded for Ag_0.05_Bi_1.95_Se_3_ single-crystal FET using a SiO_2_ gate dielectric (Fig. 1d) at 272 K. The *σ*_s_–*V*_g_ plot exhibits n-channel normally-on properties; the single crystal was 105-nm thick. Thus, the single crystals are made electron-rich without any application of *V*_g_, and the quantity of electrons in the channel region is weakly tuned by applying *V*_g_. The *σ*_s_–*V*_g_ plot at 60 K is also shown in Fig. 3a. The plot shows a steeper slope than that at 272 K when applying negative *V*_g_, implying an effective depletion of the channel region when applying negative *V*_g_. These results suggest the presence of significant bulk conductance even in Ag_0.05_Bi_1.95_Se_3_. This problem is discussed later. Figure 3b shows the *ρ*_s_–*T* plots at different *V*_g_ values of −50, 0, and 50 V, with 105-nm thick Ag_0.05_Bi_1.95_Se_3_. Three *ρ*_s_–*T* plots show no difference at 200 K, but the difference increases near *T*_cr_ (=35 K), and the *ρ*_s_ decreases with increasing *V*_g_ from −50 to 50 V, consistent with the *σ*_s_–*V*_g_ plot at 60 K (Fig. 3a). The difference disappears below *T*_cr_, implying a small gate-voltage modulation of transport properties.

The schematic representation of the Ag_0.05_Bi_1.95_Se_3_ single-crystal FET with an ionic liquid gate capacitor (105-nm thick Ag_0.05_Bi_1.95_Se_3_ single-crystal electric-double-layer transistor (EDLT)) is shown in Fig. 3c. Figure 3d shows the *σ*_s_–*V*_g_ plot at 242 K for the Ag_0.05_Bi_1.95_Se_3_ EDLT, which exhibited n-channel normally-on properties and clearly showed low-voltage operation.

**Figure 4:**
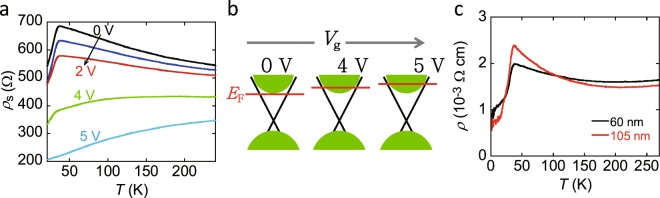
(**a**) *ρ*_s_–*T* plots of Ag_0.05_Bi_1.95_Se_3_ single crystal EDLT; thickness of single crystal is 105 nm. The applied *V*_g_ is in 0–5 V. Transport properties are measured in two-terminal measurement mode. (**b**) Schematic representations of electronic structures in Ag_0.05_Bi_1.95_Se_3_ single crystal at different *V*_g_ values. (**c**) *ρ*–*T* plots of 60-nm and 105-nm thick Ag_0.05_Bi_1.95_Se_3_ single crystals, which are measured in four-terminal measurement mode.

Figure 4a shows the *ρ*_s_–*T* plots for the Ag_0.05_Bi_1.95_Se_3_ single-crystal EDLT over a *V*_g_ range from 0 to 5 V with a 105-nm thick single crystal. The *ρ*_s_ value decreased with an increase in *V*_g_, and non-metallic behavior (an inverse V-shaped *ρ*_s_–*T* plot) at 0–2 V changed very markedly to metallic behavior at 5 V. The transition of the *ρ*_s_–*T* plot observed around 35 K becomes ambiguous with increasing *V*_g_. The *ρ*_s_–*T* plot at *V*_g_ = 5 V is similar to that of non-doped Bi_2_Se_3_ (see Fig. 2a), indicating that the Fermi level reaches the conduction band as shown in Fig. 4b. Clearly, the applied gate-voltage tunes the Fermi level.

The field-effect mobility, *μ*, of the Ag_0.05_Bi_1.95_Se_3_ single-crystal FET with a SiO_2_ gate dielectric was found to be 35(2) cm^2^ V^−1^ s^−1^ at 272 K. The *μ* value was slightly suppressed by Ag doping, as the reported lower limit of *μ* for a non-doped Bi_2_Se_3_ single crystal FET with a SiO_2_ gate dielectric^30^ is 50–100 cm^2^ V^−1^ s^−1^. The *μ* value in an Ag_0.05_Bi_1.95_Se_3_ single crystal EDLT was 40(1) cm^2^ V^−1^ s^−1^, which is smaller by an order of magnitude than the 692 cm^2^ V^−1^ s^−1^ reported in a non-doped Bi_2_Se_3_ single-crystal EDLT^31^, indicating the suppression of *μ* due to scattering by Ag doping.

## Discussion

The inverse V-shaped *ρ*–*T* (or *ρ*_s_–*T*) plot in Ag_x_Bi_2−x_Se_3_ is the most interesting observation in this study. As illustrated in Fig. 2e, the Fermi level of Ag_x_Bi_2−x_Se_3_ is located on the bottom area of the conduction band or near the top of the surface states. Therefore, the *ρ*–*T* plot should reflect the surface states to some extent, in which the density of states (DOS) is much smaller than in the conduction band. In Ag_0.05_Bi_1.95_Se_3_, the bulk region of crystal is an insulator-like and the surface is metallic, which is different from that of non-doped Bi_2_Se_3_ with the complete crossing of the Fermi level to conduction band. This situation should lead to insulating behavior at high temperatures, because the bulk contribution may predominate for thick crystal and the transport property should be governed by the thermal excitation to the conduction band. In other words, the surface transport is hidden by the bulk conduction. This is the first scenario for explaining the insulating behavior above *T*_cr_.

Here, it should be noticed that the temperature dependence of *ρ* reflecting surface states may slowly decrease at low temperature, because the *ρ*–*T* plot of non-doped graphene, which were measured with/without applying *V*_g_, showed a very slow decrease with decreasing temperature below 150 K^32,33^. Therefore, the rapid decrease in *ρ*–*T* plot below *T*_cr_ (=35 K) found for Ag_x_Bi_2−x_Se_3_ may not be explained by considering the conduction through the surface states. Therefore, we assumed that the Fermi level shifted upward to cross the conduction band at *T*_cr_. In this case, the bulk transport should suddenly change from insulating to metallic behaviour. If it is the case, the inverse-V shaped *ρ*–*T* plot mainly reflects a bulk transport in a whole temperature range (1.5–270 K), although the surface states indirectly affects the *ρ*–*T* plot. The *n* value determined from Hall effect (Fig. 2c) shows the drastic increase with decreasing temperature below *T*_cr_, which correlates with *ρ*–*T* plot, showing that the variation of *ρ* can be substantially explained by electron density. Such a drastic increase in *n* may be easily explained by the upward moving of the Fermi level. However, it is still unclear whether such a Fermi level shift takes place below the *T*_cr_, and why it happens. We must investigate whether the structural transition or the electronic variation occurs at *T*_cr_, using various probe techniques such as temperature-dependent XRD, temperature dependent X-ray emission spectroscopy and so on.

We may comment on the other scenario for insulating behaviour of *ρ*–*T* plot in the high-temperature range above the *T*_cr_. The insulating behaviour in Ag_x_Bi_2−x_Se_3_ may originate from the crossing of the Fermi level to surface states, since the defective graphene provides an insulating behaviour for the *ρ*–*T* plot^34^. If the surface region of Ag_x_Bi_2−x_Se_3_ will be much defective, the insulating behaviour may be observed in the *ρ*–*T* plot. This is the second scenario. Even in this scenario, we must assume the Fermi level shift for explaining the *ρ*–*T* plot below the *T*_cr_.

Figure 4c shows the *ρ*–*T* plots from 60-nm and 105-nm thick Ag_0.05_Bi_1.95_Se_3_ single crystals. The slope of the temperature dependence curve of *ρ* in thick single crystal (105 nm) both above and below *T*_cr_ is steeper than that of thin single crystal (60 nm). In case of thick single crystal, the bulk transport should contribute significantly to the transport properties in comparison with thin single crystal. Therefore, the change of transport properties between thin and thick single crystals suggests that the *ρ*–*T* plot may mainly reflect the bulk transport in all temperature range, since the surface states should provide a constant contribution to the *ρ*–*T* plot regardless of thickness of crystal. As a consequence, we insist that the first scenario may explain the inverse-V shaped *ρ*–*T* plots observed in this study, although the second scenario must be more fully examined.

## Conclusion

This study reports the FET properties as well as transport property and Hall effect of single crystals of Ag doped Bi_2_Se_3_. The Ag doping of Bi_2_Se_3_ provided a drastic change of transport property from metallic behavior in non-doped Bi_2_Se_3_, *i.e*., the characteristic inverse V-shaped *ρ*– *T* plot was observed in Ag_x_Bi_2−x_Se_3_ (x = 0.05 and 0.2). This behavior should be due to the successful tuning of the Fermi level downward through hole doping, because Ag is substituted for Bi in Ag_x_Bi_2−x_Se_3_ (x = 0.05 and 0.2), although the intercalation of Ag to Bi_2_Se_3_ lattice may also take place for x = 0.2 in addition to the substitution of Ag for Bi. The temperature-dependent Hall effect measurement showed that the *ρ* – *T* plot correlated with the *n*–*T* plot, *i.e*., the electron density plays an important role for the *ρ*–*T* plot. Two different scenarios were suggested to explain the inverse V-shaped *ρ*–*T* plot. As a consequence, we insisted that the *ρ*–*T* plot may mainly reflect the bulk transport in all temperature range and reflect the thermal electron excitation to conduction band at high temperature, and that the Fermi level upward-shift may happen at *T*_cr_ and the metallic property is observed below *T*_cr_. The positive *V*_g_ application (or electron accumulation) for Ag_0.05_Bi_1.95_Se_3_ successfully changed the inverse V-shaped *ρ*–*T* plot to metallic one, suggesting an upward tuning of the Fermi level. Thus, this study evidenced the successful and free tuning of the Fermi level by doping of Ag atom and positive gating in Ag doped Bi_2_Se_3_ with ionic liquid. In additon, the pressure application for Sb_2_Se_3_, BiTeI and HfTe_5_ provided the variation of electric transport from semiconducting/semimetallic to metallic behavior through an instesting topological quantum transition^35–37^, which can be recognized as ‘physical tuning of the Fermi level’. Therefore, the element doping may be termed as ‘chemical tuning of the Fermi level or the Fermi surface’. Both combination of physical and chemical tuning may enable the wide tunig of the Fermi level and the Fermi surface to induce novel physical properties. To sum up, this study may provide us with a fruitful knowledge of physics of topological materials and experience for manufacturing practical transistor devices based on topological insulators.

## Methods

The crystals of Ag_x_Bi_2_Se_3_ were grown by a conventional melt-growth method using stoichiometric amounts of Ag, Bi, and Se powders. The detailed experimental process was as follows: the powders were sealed in a quartz tube which was heated at 800 °C for 12 h. It was slowly cooled down to 650 °C at the rate of 30 °C/h, and then quenched with ice-water. The obtained crystals showed a clear basal plane structure. Their crystal structures were determined by single-crystal XRD measurements, using a Rigaku Saturn 724 diffractometer equipped with a Mo Kα source (wavelength λ = 0.71073 Å); measurement was performed at 100 K. Thickness and surface morphology of single crystals were determined by AFM equipment (SII technology, SPA400-DFM). The EDX spectrum was recorded with an EDX spectrometer equipped with a scanning electron microscope (SEM) (KEYENCE VE-9800 - EDAX Genesis XM2). The AFM and EDX measurements were made at room temperature.

In the measurements of electric transport and Hall effect, the *I* was applied with a KEITHLEY-220 Programmable current source. The exact value of *I* was monitored by an Advantest R-8240 digital electrometer. The *V* was measured with an Agilent 34420 A nano-voltmeter. For Hall effect measurement, the magnetic field, *B*, was controlled with Oxford Instruments, MercuryiPS. The gate voltage (*V*_g_) was applied to a heavily doped Si substrate for the Ag_0.05_Bi_2_Se_3_ single-crystal FET with a 300-nm thick SiO_2_ gate dielectric, and to the side gate electrode (Au electrode) for the Ag_0.05_Bi_2_Se_3_ single-crystal EDLT with ionic liquid, using a KEITHLEY 2635 A sourcemeter. Device structures (top-contact type) are illustrated in Figs 1d, 2b and 3c; top-contact electrodes and side-gate electrodes were formed by 100-nm thick Au, and 5-nm thick Cr was inserted between the Au electrode and the single crystal. Temperature was controlled using an Oxford superconducting magnet system with a variable temperature insert. The ionic-liquid 1-butyl-3-methylimidazolium hexafluorophosphate (bmim[PF_6_]) was levigated by adding poly(styrene-b-ethylene oxide-b-styrene), and was placed across the side gate electrode and Ag_0.05_Bi_2_Se_3_ single crystal. All single crystals used for transport measurements were exfoliated using the scotch tape under air.

## Supplementary information

Supplementary Information
