# Supplementary material for: Fermi level tuning of Ag-doped Bi2Se3 topological insulator
Source: Sci Rep. 2019 Mar 29;9:5376. doi: 10.1038/s41598-019-41906-7 (PMC6440949; doi:10.1038/s41598-019-41906-7)
Supplement: Supplementary file 1 — Supplementary Information [file 41598_2019_41906_MOESM1_ESM.pdf]

Supplementary Information

Fermi level tuning of Ag-doped Bi_2Se_3 topological insulator

Eri Uesugi[1], Takaki Uchiyama[1], Hidenori Goto[1], Hiromi Ota[2], Teppei Ueno[1], Hirokazu Fujiwara[1], Kensei Terashima[1], Takayoshi Yokoya[1], Fumihiko Matsui[3], Jun Akimitsu[1], Kaya Kobayashi[1], and Yoshihiro Kubozono[1*]

[1]Research Institute for Interdisciplinary Science, Okayama University, Okayama 700-8530, Japan

[2]Advanced Science Research Centre, Okayama University, Okayama 700-8530, Japan

[3]Graduate School of Materials Science, Nara Institute of Science and Technology, Ikoma 630-0192, Japan

The crystallographic data of $Ag_{0.05}Bi_2Se_3$ were shown in Table S1, which were determined by single crystal XRD analyses. Bragg spots of $Ag_{0.05}Bi_{1.95}Se_3$ single crystal are shown in Figure S1.

Table S1. Crystallographic data for $Ag_{0.05}Bi_2Se_3$ and Bi_2Se_3. The Ag atom is assumed to be located at the 6*c* site occupied by Bi atom.

$Ag_{0.05}Bi_2Se_3$

Rhombohedral lattice: $R\bar{3}m$ (No. 166)

Lattice constant: a = 4.146(6) Å, c = 28.66(4) Å

		Occ.	*x*	*y*	*z*	*B* (Å)
6*c*	Bi(Ag)	1.0	0.00000	0.00000	0.40079(4)	0.73(5)
6*c*	Se	1.0	0.00000	0.00000	0.2105(1)	0.66(6)
3*a*	Se	1.0	0.00000	0.00000	0.00000	0.72(8)

Bi_2Se_3[a)]

Rhombohedral lattice: $R\bar{3}m$ (No. 166)

Lattice constant: a = 4.18 Å, c = 28.7 Å

		Occ.	*x*	*y*	*z*
6c	Bi	1.0	0.00000	0.00000	0.40046
6c	Se	1.0	0.00000	0.00000	0.2097
3*a*	Se	1.0	0.00000	0.00000	0.00000

a) Crystallographic data for Bi_2Se_3 was taken from Ref. 23.

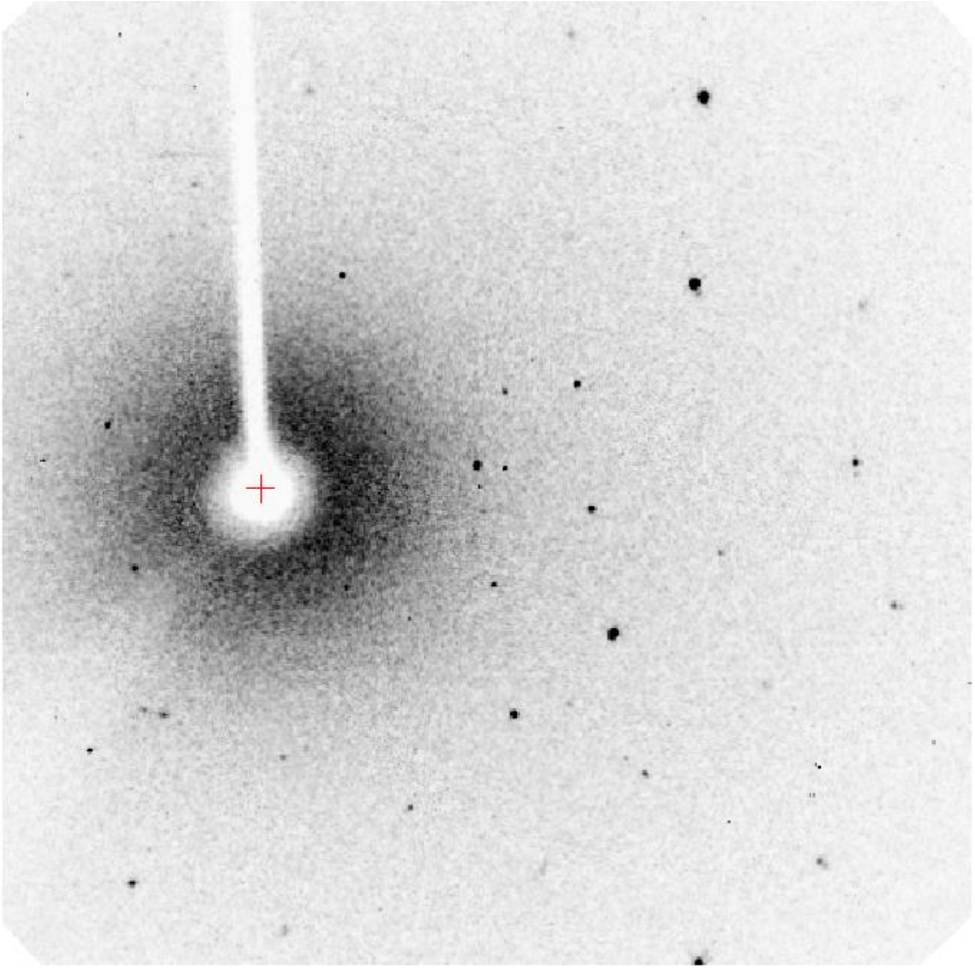

Figure S1. Bragg spots of $Ag_{0.05}Bi_{1.95}Se_3$ single crystal.
